# Lifestyle intervention outcomes in T2DM and the added value of CGM: 6-month results from the Polish cohort of the C4D study

**DOI:** 10.1038/s41598-026-55083-x

**Published:** 2026-05-25

**Authors:** Joanna Ostrowska, Ewa Kobos, Mariola Pietrzak, Agata Szymczak, Katarzyna Wiktorzak, Marta M. Pisano-González

**Affiliations:** 1 Department of Clinical Dietetics, Faculty of Health Sciences, Medical University of Warsaw, E Ciołka 27, Warsaw, 01-445 Poland; 2 Department of Development of Nursing, Social and Medical Sciences, Faculty of Health Sciences, Medical University of Warsaw, Żwirki i Wigury 61, Warszawa, 02-091 Poland; 3 Office of Public Partnership and Innovation, National Health Fund Headquarters, ul. Rakowiecka 26/30, Warsaw, 02-528 Poland; 4 Ministry of Health of the Principality of Asturias (CSPA), Calle Ciriaco Miguel Vigil, 9, Oviedo, 33005 Spain; 5 Health Research Institute of the Principality of Asturias (ISPA), Avda. Del Hospital Universitario, Oviedo s/n, 33011 Spain

**Keywords:** Type 2 diabetes, Lifestyle, Self-monitoring of blood glucose (SMBG), Continuous glucose monitoring (CGM), HbA1c, Weight loss, Diseases, Endocrinology, Health care, Medical research

## Abstract

Type 2 diabetes remains a major public health challenge, requiring lifelong management. Structured lifestyle-based interventions are increasingly recognised for their role in supporting self-management. The CARE4DIABETES (C4D) programme, part of a Joint Action funded by the EU4Health initiative, aims to implement and evaluate a behavioural lifestyle model (Reverse Diabetes2 Now) across 12 European countries. C4D is a 12-month, quasi-experimental, structured, digitally supported lifestyle programme comprising multiple group-based educational sessions delivered by a multidisciplinary team (MDT) and addressing nutrition, physical activity, sleep, and stress management. This interim analysis includes the Polish cohort (*n* = 38; type 2 diabetes duration ≤ 10 years), presented overall and stratified by continuous glucose monitoring (CGM) users (*n* = 21) versus self-monitoring of blood glucose (SMBG, *n* = 17). At 6 months, participants showed significant improvements. Mean HbA1c decreased by 0.78% points (–10.9%; *p* < 0.001) to 6.35%. Body weight decreased by 6.04 kg (–6.5%; *p* < 0.001), waist circumference by 6.77 cm (–6.4%; *p* < 0.001), fat mass by 2.47 kg (–7.2%; *p* < 0.001), and triglycerides by 20.3% (*p* = 0.023), while total cholesterol, LDL-C, and HDL-C did not change significantly. Improvements were numerically larger in the CGM group. Between-group comparisons of change scores showed greater reductions in body weight and BMI in the CGM group compared with the SMBG group (*p* < 0.05; Cohen’s d ≈ 0.9–1.0). The 6-month intensive phase of this structured, group-based lifestyle education programme was associated with clinically meaningful improvements in glycaemic control and anthropometric outcomes. Improvements were greater among CGM users than in the SMBG group, suggesting that integrating CGM into structured education may further enhance programme outcomes.

## Introduction

Type 2 diabetes (T2DM) is the most common form of diabetes, accounting for over 90% of cases worldwide, and its prevalence continues to rise^1^. Evidence consistently shows that weight reduction and long-term weight maintenance, achieved through appropriate dietary changes and increased physical activity, are central to the management of T2DM. Such lifestyle changes improve glycaemic control and other major cardiometabolic risk factors, including blood pressure, lipid profile, and inflammatory status^2,3^.

As a chronic condition, T2DM requires daily self-management, regular monitoring of metabolic parameters, and numerous decisions made in everyday life. For this reason, patient education is a cornerstone of effective care and has long been recognised as a standard component of diabetes management. Recent recommendations from the European Association for the Study of Diabetes (EASD) and the Diabetes Nutrition Study Group (DNSG) underline the value of long-term, structured education programmes delivered by multidisciplinary teams (MDT) and grounded in behavioural therapy, as these approaches help patients understand and implement sustainable health behaviours and thereby support durable weight loss^4^. Importantly, clinically meaningful and sustained weight loss may allow dose reduction of glucose-lowering medication and, in some individuals, contribute to T2DM remission^5^. The Polish Diabetes Association (PTD) also recommends that structured education and ongoing re-education should be available to all people with T2DM and their caregivers and emphasises delivery by a qualified MDT^6^.

Education can be delivered individually or in groups, in-person, remotely, or in hybrid formats, with possible involvement of family members or caregivers. Group-based programmes led by MDTs have been linked to better metabolic outcomes, higher treatment satisfaction, and stronger motivation for lifestyle change^7,8^. Some studies suggest they may be at least as effective as individual education and, in certain settings, more effective in sustaining long term benefits^9^. Tele-education is also gaining importance through educational platforms and mobile applications, telephone and video consultations, webinars, e-learning, and SMS reminders^10^. Available evidence further suggests that tele-education may be comparable to traditional approaches and, in some analyses, was associated with a greater reduction in HbA1c (mean difference − 0.3%; 95% CI: −0.42 to − 0.19)^11,12^.

Within this context, continuous glucose monitoring (CGM) may further strengthen lifestyle education by providing detailed glucose profiles that help patients and clinicians link everyday behaviours (such as meals, physical activity, sleep, and stress) to glucose patterns. The most effective approaches are therefore likely to combine digital tools (including CGM) with structured education and behavioural support tailored to individual needs and capacities^10^. An example of such an integrated model is CARE4DIABETES (C4D), a year-long structured lifestyle programme delivered within a Joint Action involving 30 partners from 12 European Union countries (Belgium, Bulgaria, Slovakia, Slovenia, Finland, Greece, Hungary, Italy, Malta, Poland, Portugal, and Spain as the consortium lead). C4D implemented an intensive, outpatient lifestyle education programme for people with T2DM to support active self-management. The intervention is based on the Dutch programme ReverseDiabetes2Now (developed by Voeding Leeft)^13–16^, which has been implemented in the Netherlands for a decade and was recognised by the European Union as a “best practice” before being piloted in participating countries, including Poland. According to data reported by Voeding Leeft, 92% of people with T2DM achieved metabolic control after 12 months of participation, allowing partial or complete discontinuation of pharmacotherapy^13^. The programme targets four core lifestyle domains: diet, physical activity, stress management, and sleep duration and quality, and differs from usual care through its group-based structure (approximately 20 participants per group), the intensity of delivery (six full-day sessions), and its strong emphasis on therapeutic lifestyle change, with diet as a central component.

In this study, we addressed two questions. First, we assessed whether participation in a structured lifestyle intervention was associated with improvements in glycaemic control and body composition over 6 months in individuals with T2DM. Second, we evaluated whether adding CGM to the same programme was associated with greater improvements in metabolic outcomes compared with participation without CGM.

## Methods

### Study design and setting

CARE4DIABETES (C4D) is a 12-month, quasi-experimental study implemented across 12 European countries, adapted from the Dutch “Reverse Diabetes 2Now” best practice^13–16^. The study methodology has been described in detail in the published protocol^17^. In brief, C4D is a 12-month, structured lifestyle programme for adults with type 2 diabetes (up to 10 years since diagnosis), delivered by a multidisciplinary team (MDT) through multiple group-based educational sessions covering four domains: nutrition, physical activity, sleep, and relaxation/stress management^17^.

### Participants and eligibility

Participants were adults with physician diagnosed T2DM recruited from a Polish primary care clinic. Eligible patients were invited by their general practitioners (GP), who also provided clinical follow-up during the programme.

Inclusion criteria were in line with the C4D protocol^17^: adults aged 20–80 years with type 2 diabetes of 1–10 years’ duration, a BMI of 25–40 kg/m², and pharmacological treatment (oral and/or injectable agents, including insulin). Use of GLP-1 receptor agonists or SGLT-2 inhibitors required a minimum of 6 months on a stable dose/regimen prior to programme start. Further eligibility requirements included the absence of major contraindications to participation (e.g., COPD, heart failure, severe renal impairment defined as eGFR < 30 mL/min/1.73 m², prior bariatric surgery, eating disorders, or pregnancy), willingness to engage in lifestyle change, adequate language proficiency, access to and ability to use an internet-enabled device, and willingness to perform home blood glucose monitoring. Willingness to engage in lifestyle change was assessed by the recruiting GP during the eligibility visit and was based on the patient’s declared readiness to participate in the full programme, attend group sessions, implement lifestyle recommendations, perform home glucose monitoring, and use the digital platform. Adequate language proficiency was defined as sufficient Polish language comprehension to understand study information, provide informed consent, communicate with the MDT, and participate in group education without translation support.

In Poland, 38 participants were enrolled. All participants took part in the same structured lifestyle intervention, delivered through educational sessions in two cohorts of similar size. One cohort used CGM (*n* = 21), whereas the other used standard SMBG with a glucometer (*n* = 17), in line with instructions provided by the physician and nurse within the MDT. The implementation was conducted as a pragmatic, real-world lifestyle programme, with non-randomized allocation to the CGM or SMBG group. Participants were assigned to consecutive cohorts according to the timing of enrolment, with no individual prescription or clinical selection of the monitoring method by the study GP or nurse. The only intended difference between cohorts was the glucose monitoring method used.

### Intervention

C4D is a structured education programme grounded in behavioural change principles (I-Change model)^18^ and designed to help participants practise and integrate new habits rather than only receive information. The programme lasts 12 months and includes two consecutive stages: (I) a 6-month intensive training phase and (II) a 6-month aftercare phase. The 6-month intensive phase consisted of six full-day, structured group sessions delivered by the MDT (GP/diabetologist, dietitian, psychologist, and nurse) and focused on key behavioural therapy components: diet, physical activity, stress coping, and healthy sleep.

In Poland, sessions were delivered in a hybrid format: four face-to-face workshops and two remote (telematic) workshops. Workshops took place at programme start (kick-off meeting with an overnight stay), and then at months 1 (remote), 3 (face-to-face), 4.5 (remote), and 6 (face-to-face). A distinctive feature of the in-person component was an overnight kick-off meeting. The aim was to build group cohesion and encourage early engagement by removing participants from their usual routines, reducing exposure to triggers (e.g., habitual snacking), and providing an added layer of support (particularly for those using medications associated with hypoglycaemia).

Key components of the intervention are described in the published protocol^17^. In brief, the programme combines education with practical activities. Sessions covered healthy eating principles, meal planning and preparation, cooking classes, food label reading, practical recipe modification, supervised physical exercises, relaxation techniques, and basic coaching practices. Behavioural support was embedded throughout, using group work and peer support to maintain motivation and facilitate adherence. Participants also received introductory education on T2DM pathophysiology, interpretation of daily glucose patterns, biometric feedback, and practical self-management strategies. The entire intervention was supported by a digital platform enabling peer interaction and contact with the MDT, facilitating ongoing coaching and medical monitoring. Throughout the programme, participants were supervised by the MDT, and medication adjustments were made when clinically indicated.

As described above, one group used CGM, and the second group followed SMBG. Both cohorts received the same intervention content, followed the same session structure and educational materials, and were supported by the same MDT framework, including a GP/diabetologist, dietitian, psychologist, and nurse. The only planned differences between cohorts were the glucose monitoring method used and the timing of programme delivery, as the cohorts were delivered in separate but partly overlapping time periods. No changes were made to the intervention content, educational materials, or session structure between cohorts.

In the CGM group, FreeStyle Libre 2 sensors (Abbott Diabetes Care) were applied according to a predefined schedule: two 14-day sensors at baseline, one 14-day sensor at 3 months, and one 14-day sensor at 6 months of the lifestyle intervention. Outside these CGM periods, participants measured capillary blood glucose using glucometers, following instructions provided by the physician and nurse within the MDT.

### Data collection

Clinical, anthropometric, and body composition measures were collected by the MDT using standardised procedures at baseline and at 6 months, in line with the protocol measurement schedule^17^. Although the protocol also includes an assessment at 12 months, the present paper reports interim outcomes for the Polish cohort based on baseline and 6-month data only.

Body weight and height were measured using a Seca 799 measuring station (column scale) with a precision of ± 0.1 kg and ± 0.1 cm, respectively. Waist circumference was measured using a steel tape positioned midway between the lower rib margin and the iliac crest, in the horizontal plane. Body composition was assessed using bioelectrical impedance analysis (BIA) with a multi-frequency segmental analyzer (Tanita MC-780MA, Tanita Corporation, Tokyo, Japan) following the manufacturer’s instructions. Participants were asked to follow the European Society for Clinical Nutrition and Metabolism (ESPEN) recommendations^19^. Specifically, they were instructed to fast for at least 2–3 h before testing, avoid physical activity for 12 h, abstain from alcohol and caffeinated or carbonated beverages for 24 h, and empty their bladder 30 min prior to the assessment. Biochemical measurements included HbA1c (%), total cholesterol (mg/dL), HDL cholesterol (mg/dL), LDL cholesterol (mg/dL), and triglycerides (mg/dL).

### Statistical analysis

Analyses were performed in participants with paired measurements at baseline and 6 months. Continuous variables are presented as mean ± SD. Baseline differences between CGM and SMBG groups were assessed using t-tests or Mann-Whitney U tests for continuous variables and Fisher’s exact test for categorical variables. Within-participant changes were evaluated using paired t-tests; normality of the change scores was assessed (Shapiro–Wilk test), and when the normality assumption was not met, the Wilcoxon signed-rank test was used as a sensitivity analysis. Two-sided p-values < 0.05 were considered statistically significant. Effect sizes were quantified using Cohen’s d for paired data, and 95% confidence intervals for mean changes were calculated using the t distribution. Participants were additionally stratified by glucose monitoring method (CGM vs. SMBG). Between group differences in baseline to 6-month change scores (ΔCGM vs. ΔSMBG) were evaluated using an independent-samples t-test; when distributional assumptions were not met, the Mann–Whitney U test was used as a sensitivity analysis. Between-group effect sizes were expressed as Cohen’s d for independent groups (based on change scores) and interpreted as small (|d| < 0.2), medium (0.2 ≤ |d| < 0.5), large (0.5 ≤ |d| < 0.8), and very large (|d| ≥ 0.8). Because multiple outcomes were analysed, p-values were interpreted descriptively without adjustment for multiple comparisons.

### Ethics

The Polish implementation received approval from the Bioethics Committee at the Medical University of Warsaw (No. KB/270/2023, 13 November 2023). All methods were performed in accordance with relevant guidelines and regulations, and the study was conducted in accordance with the Declaration of Helsinki. All participants received a participant information sheet and provided written informed consent prior to participation.

## Results

A total of 38 participants were included in the baseline analysis (CGM, *n* = 21; SMBG, *n* = 17). The cohort was predominantly female (63.2%), with a mean age of 56.13 ± 11.17 years. At baseline, mean body weight was 95.45 ± 16.61 kg and mean BMI was 32.06 ± 4.73 kg/m², corresponding to obesity on average, while mean waist circumference was 106.71 ± 10.97 cm, indicating substantial central adiposity. Mean HbA1c was 7.20 ± 1.67% - above commonly used glycaemic targets for adults with type 2 diabetes. Lipid parameters were as follows: total cholesterol 179.45 ± 39.76 mg/dl, HDL cholesterol 47.17 ± 11.36 mg/dl, LDL cholesterol 104.38 ± 37.55 mg/dl, and triglycerides 163.68 ± 78.95 mg/dl. Baseline anthropometric, body composition and metabolic characteristics for the total sample and by subgroup are summarised in Table 1. Descriptively, the CGM group tended to have higher adiposity parameters than the SMBG group, with statistically significant differences in body weight, BMI, fat mass, and visceral fat, while waist circumference, HbA1c, fat-free mass, muscle mass, total body water, and lipid parameters did not differ significantly between groups.

**Table 1:** Baseline characteristics of the study group (*n* = 38).

Parameter	All (Baseline) (M ± SD / *n* (%))	CGM (Baseline) (M ± SD / *n* (%))	SMBG (Baseline) (M ± SD / *n* (%))	*p*-value (between groups)
**N**	38	21	17	-
**Sex: female**,** n (%)**	24 (63.2%)	14 (66.7%)	10 (58.8%)	0.740
**Sex: male**,** n (%)**	14 (36.8%)	7 (33.3%)	7 (41.2%)	0.740
**Age (years)**	56.13 ± 11.17	57.0 ± 12.1	55.06 ± 10.15	0.601
**Body weight (kg)**	95.45 ± 16.61	101.39 ± 17.78	88.12 ± 11.8	0.012*
**Height (m)**	1.7 ± 0.09	1.71 ± 0.09	1.69 ± 0.09	0.371
**BMI (kg/m²)**	32.06 ± 4.73	33.55 ± 5.26	30.93 ± 3.1	0.017*
**Waist circumference (cm)**	106.71 ± 10.97	108.95 ± 11.73	103.94 ± 9.58	0.297
**Fat mass (kg)**	36.21 ± 11.14	39.63 ± 11.19	31.98 ± 9.81	0.033*
**Visceral fat**	13.39 ± 4.3	14.71 ± 4.42	11.76 ± 3.63	0.033*
**FFM (kg)**	60.1 ± 11.01	61.66 ± 11.95	58.17 ± 9.73	0.339
**Muscle mass (kg)**	57.09 ± 10.5	58.57 ± 11.39	55.26 ± 9.28	0.340
**Total body water**	44.59 ± 6.16	44.49 ± 5.71	44.72 ± 6.83	0.913
**HbA1c (%)**	7.2 ± 1.67	7.67 ± 1.94	6.62 ± 1.06	0.064
**Total cholesterol (mg/dl)**	179.45 ± 39.76	176.33 ± 40.63	183.29 ± 39.55	0.598
**HDL cholesterol (mg/dl)**	47.17 ± 11.36	46.59 ± 9.69	47.88 ± 13.41	0.803
**LDL cholesterol (mg/dl)**	104.38 ± 37.55	100.88 ± 40.03	108.71 ± 34.95	0.530
**Triglycerides (mg/dl)**	163.68 ± 78.95	149.19 ± 56.74	181.59 ± 98.85	0.649

* *p* < 0.05.

Out of the 38 participants enrolled in Poland, 31 completed the 6-month intensive phase and had paired baseline and 6-month data available for analysis, corresponding to an overall completion rate of 81.6%. Completion rates were similar in the CGM and SMBG groups: 17 of 21 participants in the CGM group (81.0%) and 14 of 17 participants in the SMBG group (82.4%) completed the 6-month assessment. The main reasons for attrition were withdrawal from participation, loss to follow-up, and personal or organisational reasons. However, detailed reasons were not systematically collected.

Among participants with paired baseline and 6-month data, glycaemic control improved significantly: mean HbA1c decreased from 7.13% to 6.35% (mean change − 0.78% points; *p* < 0.001; large effect). In parallel, participants achieved clinically meaningful reductions in anthropometric outcomes, with an average weight loss of 6.0 kg and a waist circumference reduction of 6.8 cm (both *p* < 0.001), alongside a significant decrease in BMI (− 2.11 kg/m²; *p* < 0.001; very large effects for weight-related outcomes).

Body composition analyses indicated a significant decrease in fat mass (− 2.47 kg; *p* < 0.001) and a reduction in visceral fat (− 0.93; *p* = 0.001). However, reductions were also observed in fat-free mass (− 1.89 kg) and estimated muscle mass (− 2.19 kg) (both *p* < 0.001), suggesting that weight loss was accompanied by losses in lean tissue. Total body water percentage did not change significantly (*p* = 0.153). In the lipid profile, triglycerides decreased significantly (− 34.9 mg/dL; *p* = 0.023), whereas changes in total cholesterol and LDL cholesterol were modest and did not reach statistical significance; HDL cholesterol remained stable. Detailed results are presented in Table 2.

**Table 2:** Changes in metabolic and anthropometric parameters after 6-month lifestyle intervention (*n* = 31).

Parameter	Baseline (M ± SD)	6-month (M ± SD)	Change (M ± SD)	95% CI for Change	Change (%)	*p*-value	Sig.	Cohen’s d	Effect size
**HbA1c (%)**	7.13 ± 1.68	6.35 ± 1.13	-0.78 ± 1.06	(-1.17, -0.39)	-10.9%	< 0.001	***	-0.73	large
**Body weight (kg)**	92.89 ± 15.81	86.85 ± 14.6	-6.04 ± 4.69	(-7.76, -4.32)	-6.5%	< 0.001	***	-1.29	very large
**BMI (kg/m²)**	32.06 ± 4.34	29.94 ± 3.75	-2.11 ± 1.65	(-2.72, -1.51)	-6.6%	< 0.001	***	-1.28	very large
**Waist circumference (cm)**	105.74 ± 10.75	98.97 ± 9.45	-6.77 ± 4.51	(-8.43, -5.12)	-6.4%	< 0.001	***	-1.5	very large
**Fat mass (kg)**	34.44 ± 10.48	31.97 ± 9.51	–2.47 ± 3.02	(-3.62, -1.32)	–7.2%	< 0.001	***	–0.82	very large
**Visceral fat**	13.45 ± 4.13	12.52 ± 4.01	-0.93 ± 1.41	(-1.47, -0.39)	-6.9%	0.001	**	-0.66	large
**FFM (kg)**	59.50 ± 11.31	57.61 ± 11.48	–1.89 ± 2.06	(-2.67, -1.1)	–3.2%	< 0.001	***	–0.92	very large
**Muscle mass (kg)**	56.52 ± 10.79	54.33 ± 11.31	–2.19 ± 2.69	(-3.21, -1.17)	–3.9%	< 0.001	***	–0.81	very large
**Total body water (%)**	44.65 ± 5.95	45.49 ± 5.93	0.84 ± 3.10	(-0.33, 2.02)	+ 1.9%	0.153	ns	0.27	medium
**Triglycerides (mg/dl)**	171.48 ± 82.09	136.61 ± 56.81	-34.87 ± 80.96	(-64.57, -5.18)	-20.3%	0.023	*	-0.43	medium
**Total cholesterol (mg/dl)**	176.1 ± 39.46	167.03 ± 46.35	-9.06 ± 26.63	(-18.83, 0.7)	-5.1%	0.068	ns	-0.34	medium
**LDL cholesterol (mg/dl)**	100.75 ± 40.03	94.69 ± 39.06	-6.06 ± 24.7	(-15.12, 3.0)	-6.0%	0.182	ns	-0.25	medium
**HDL cholesterol (mg/dl)**	46.59 ± 11.59	46.16 ± 10.59	-0.43 ± 7.07	(-3.03, 2.16)	-0.9%	0.736	ns	-0.06	small

* *p* < 0.05; ** *p* < 0.01; *** *p* < 0.001.

Table 3 summarizes changes in metabolic and anthropometric parameters after 6 months in 31 participants who completed a 6-month group-based structured educational programme, stratified into two groups: participants using CGM (*n* = 17) and participants using SMBG (*n* = 14). Overall, both groups showed improvements in metabolic control and reductions in weight-related measures. The largest between-group differences were observed for body weight and BMI, with greater reductions in the CGM group compared with the SMBG group (between-group comparison of change significant for both outcomes). For most other outcomes (including HbA1c, waist circumference, lipid profile, and body composition measures), changes were generally favourable in both groups; however, between-group differences were smaller and mostly did not reach statistical significance, and effect sizes ranged from small to very large depending on the outcome. Notably, changes were generally more pronounced in the CGM group than in the SMBG group, although this pattern was not consistent for all lipid outcomes.

**Table 3:** Changes in metabolic and anthropometric parameters after 6 months (CGM *n* = 17 vs. SMBG *n* = 14).

Parameter	CGM Baseline (M ± SD)	CGM 6 mo (M ± SD)	Δ CGM (*p*-value)	SMBG Baseline (M ± SD)	SMBG 6 mo (M ± SD)	Δ SMBG (*p*-value)	*p*-value (between groups)	Cohen’s d	Effect Size
HbA1c (%)	7.6 ± 2.0	6.6 ± 1.4	-1.0 ± 1.1 **	6.6 ± 1.0	6.0 ± 0.6	-0.6 ± 1.0 ns	0.296	-0.38	Medium
Body weight (kg)	98.6 ± 16.7	90.6 ± 14.8	-7.9 ± 4.1 ***	86.0 ± 11.8	82.2 ± 13.5	-3.8 ± 4.5 **	0.012 *	-0.97	Very large
BMI (kg/m²)	33.6 ± 4.8	30.8 ± 3.8	-2.7 ± 1.5 ***	30.2 ± 2.9	28.8 ± 3.5	-1.4 ± 1.6 **	0.019 *	-0.90	Very large
Waist circumference (cm)	108.4 ± 10.9	101.0 ± 8.9	-7.4 ± 4.1 ***	102.5 ± 10.0	96.5 ± 9.9	-6.0 ± 5.0 ***	0.395	-0.31	Medium
Fat mass (kg)	38.4 ± 10.1	35.8 ± 9.1	-2.6 ± 3.2 **	30.2 ± 9.4	27.8 ± 8.4	-2.4 ± 3.0 *	0.884	-0.05	Small
Visceral fat	15.1 ± 3.8	13.9 ± 3.9	-1.3 ± 1.6 ***	11.6 ± 3.8	11.1 ± 3.8	-0.6 ± 1.1 ns	0.190	-0.50	Large
FFM (kg)	61.6 ± 12.2	59.2 ± 12.1	-2.4 ± 1.9 ***	57.2 ± 10.2	55.9 ± 11.0	-1.3 ± 2.1 *	0.145	-0.56	Large
Muscle mass (kg)	58.5 ± 11.6	56.2 ± 11.5	-2.3 ± 1.9 ***	54.4 ± 9.8	52.3 ± 11.1	-2.0 ± 3.4 *	0.782	-0.10	Small
Triglycerides (mg/dl)	152.6 ± 56.8	139.1 ± 54.5	-13.6 ± 60.5 ns	194.4 ± 102.7	133.6 ± 61.4	-60.7 ± 96.5 *	0.108	0.60	Large
Total cholesterol (mg/dl)	168.8 ± 38.1	156.4 ± 43.3	-12.4 ± 17.4 **	184.9 ± 40.6	179.9 ± 48.2	-5.0 ± 35.1 ns	0.450	-0.28	Medium
LDL cholesterol (mg/dl)	93.5 ± 42.6	85.1 ± 36.8	-8.4 ± 17.0 ns	109.6 ± 36.3	106.4 ± 39.8	-3.2 ± 32.2 ns	0.565	-0.21	Medium
HDL cholesterol (mg/dl)	45.3 ± 10.2	45.8 ± 10.2	0.5 ± 7.2 ns	48.1 ± 13.3	46.6 ± 11.4	-1.6 ± 7.0 ns	0.428	0.29	Medium

*p* < 0.05; ** *p* < 0.01; *** *p* < 0.001.

## Discussion

This study evaluated a 6-month lifestyle intervention in individuals with T2DM, with analyses stratified by glucose monitoring method (CGM vs. glucometer-based SMBG). At baseline, the study population had a mean BMI in the obese range and a mean HbA1c level above the general therapeutic target for T2DM recommended by the Polish Diabetes Association (PTD)^6^. After 6 months of participation in the C4D programme, glycaemic control improved in a statistically significant and clinically meaningful manner, as reflected by a reduction in mean HbA1c. HbA1c remains the key marker for assessing glycaemic control in type 2 diabetes and has a well-established predictive value for diabetes related complications^20–22^. In the overall study group, HbA1c decreased by 0.78% points to 6.35%, which is consistent with the general PTD treatment target of ≤ 7%^6^. These results are consistent with evidence from other structured lifestyle programmes. The ReverseDiabetes2Now programme implemented in the Netherlands, which informed the design of C4D, also reported improvements in glycaemic outcomes^13^. Other lifestyle programmes have likewise reported marked metabolic benefits, including substantial weight loss, improved glycaemic control, and, in some cases, diabetes remission. In the DiRECT trial, 24% of participants lost at least 15 kg and 46% achieved remission at 12 months^23^. In the Virta Health programme, HbA1c decreased from 7.6% to 6.3% after 1 year, alongside a mean weight loss of 13.8 kg^24^. Further support comes from a lifestyle programme delivered in California (USA), where HbA1c decreased progressively over 24 weeks and remission was reported in 8% of participants^25^. Consistent reductions in HbA1c after 6 months of intervention have also been reported in studies conducted in Spain^26^, Taiwan^27^, and Lebanon^28^.

Over 6 months, the reduction in HbA1c was larger in the CGM group than in the SMBG group, however, the between-group difference did not reach statistical significance, which may partly reflect the limited sample size. These findings align with available evidence suggesting that CGM can be a useful tool to support education and lifestyle decision making in T2DM, both in dietary interventions and in multicomponent lifestyle programmes, with potential benefits for glycaemic outcomes and body weight^29^. In the randomised Steno2Tech study, CGM use among insulin-treated individuals with type 2 diabetes was associated with greater improvements in time in range (TIR), HbA1c, and BMI compared with standard glucometer-based SMBG^30^. Improved glycaemic control has also been reported with CGM use in people with type 2 diabetes who are not treated with insulin^31,32^.

The study demonstrated significant reductions in body weight (approximately 6 kg on average), waist circumference (about 6.8 cm), and BMI (− 2.11 kg/m²) over the 6-month intervention period. These findings are consistent with results from other programmes combining dietary modification and physical activity, including the SLIMMER programme, where favourable changes in body weight, diet quality, and physical activity were shown to persist during follow-up after the intervention ended^33^. A weight loss of 5–7% is generally considered clinically meaningful and is typically associated with improved glycaemic control^10,34^. Evidence from observational and interventional studies further suggests that greater reductions in adiposity, including body fat percentage, waist circumference, and BMI, may translate into larger improvements in HbA1c^35,36^. An association between higher BMI and poorer glycaemic control has also been reported in population-based analyses^37^.

In the subgroup analyses, greater reductions in body weight and BMI were observed among participants using CGM. It may be hypothesised that immediate feedback on glucose levels facilitated dietary adjustments and increased engagement in lifestyle change^10,38^. Diet and physical activity are central to maintaining glycaemic homeostasis, and CGM may strengthen patients’ capacity for self-management by providing real-time feedback and helping to reduce glucose variability^39^. In line with this, a systematic review reported that, compared with control conditions, CGM use was associated with behavioural changes, including longer daily physical activity time and lower daily energy and carbohydrate intake^40^. Available evidence also suggests that, in people with type 2 diabetes, CGM can support tighter glycaemic control compared with SMBG^31^, and CGM-based feedback has shown a moderate beneficial effect on glycaemic outcomes when used to support behaviour change in adults with and without diabetes^41^. By visualising glucose values as continuous curves, CGM can help patients understand the relationships between diet, physical activity, other lifestyle factors, and glycaemic outcomes, which may contribute to greater perceived self-efficacy in behaviour change^42^.

In this study, we observed significant reductions from baseline in both FFM and estimated muscle mass in participants using CGM as well as those using SMBG. Such changes may accompany weight loss, particularly when dietary protein intake is insufficient and resistance training is not performed regularly^43^. Epidemiological data indicate that the prevalence of sarcopenia is approximately threefold higher in people with diabetes than in those without diabetes, and in type 2 diabetes it has been estimated at around 18%^44^. Therefore, teams delivering lifestyle interventions should include an assessment of sarcopenia risk, particularly in older adults, in those with lower BMI (which in this context may reflect lower muscle mass reserves), higher HbA1c, and longer diabetes duration. In practice, this highlights the importance of adequate protein intake and incorporating resistance exercises as key components to reduce the risk of muscle mass loss during weight reduction^45,46^. In addition, the reduction in FFM was consistent with the overall decrease in body weight and abdominal circumference observed during the intervention. Nevertheless, because BIA-derived estimates of FFM depend largely on total body water, some influence of hydration status cannot be excluded, even under carefully standardised measurement conditions in line with ESPEN recommendations^19,47,48^.

Regarding lipid parameters, a significant improvement was observed only for triglycerides. Similar findings were reported by Pot et al., where plasma lipid concentrations remained largely unchanged after a 6-month lifestyle intervention, except for a reduction in triglycerides^49^. Meta-analytic evidence also indicates that weight reduction is one of the key factors associated with lowering triglyceride levels^50^. The absence of significant improvement in HDL-C and LDL-C in the present study may appear unexpected, given that the intervention included both dietary and physical activity components. Physical activity may favourably influence HDL-C in people with T2DM, partly through improved insulin sensitivity and reductions in triglyceride concentrations, while post-meal exercise may support better glycaemic control and reduce postprandial lipaemia^51–54^. Previous studies also suggest that different types of exercise and multicomponent exercise interventions may improve glycaemic and lipid metabolism, including HDL-C, LDL-C, and triglycerides, in people with T2DM^55–57^. In addition, longer term follow-up may be needed to detect some lipid changes. In the study by Pot et al., significant improvements in triglycerides, the total cholesterol to HDL-C ratio, and HDL-C were observed 24 months after the start of the 6-month intervention, whereas total cholesterol did not differ significantly from baseline^58^. In the present study, however, detailed data on daily saturated fat intake, meal composition, and the timing or intensity of physical activity were not systematically collected. Therefore, we could not determine whether these factors contributed to the lack of significant HDL-C and LDL-C changes. The absence of significant changes in total cholesterol and LDL-C may also be related to concomitant use of lipid lowering therapy, which could have limited the potential to detect additional intervention effects.

Finally, it is worth highlighting the group-based structure of the intervention. Participants took part in group education delivered by an MDT including a GP/diabetologist, a dietitian, a psychologist, and a nurse. Evidence comparing individual and group education in people with T2DM suggests that group-based approaches can yield favourable outcomes, including improvements in HbA1c, body weight, waist circumference, triglycerides, and diabetes related knowledge. Moreover, interventions delivered with a group component have more often been associated with greater HbA1c improvement than those implemented without peer-group support^59,60^. At the same time, available evidence indicates that effects may be stronger when CGM is combined with behavioural or educational interventions, resulting in greater improvements in glycaemic and psychosocial outcomes than CGM alone or education alone^61^. Similarly, combining diabetes self-management education and support (DSMES) with intermittently scanned CGM (isCGM) has been associated with a larger reduction in HbA1c compared with DSMES alone^62^. Taken together, these findings suggest that periodic use of CGM as an adjunct to group-based education may enhance the effectiveness of lifestyle-focused interventions.

This study has several limitations. First, allocation to the glucose monitoring method was not randomized. Participants were assigned to consecutive cohorts according to the timing of enrolment as part of a pragmatic, real-world lifestyle programme. Although there was no intentional selection of participants for CGM or SMBG based on clinical characteristics, the cohort-based allocation may have contributed to baseline differences between groups. The CGM group had significantly higher baseline values for several adiposity parameters, which may have influenced between-group comparisons of change scores. Similarly, because the CGM and SMBG groups participated in the programme as separate cohorts, minor differences related to group dynamics, timing, or session delivery cannot be excluded, although the same structured content and MDT framework were used. In addition, outcomes were compared between participants using CGM and SMBG without a parallel control group receiving usual care, which makes it difficult to attribute the observed changes solely to the intervention. Another limitation is the 6-month follow-up, which does not allow assessment of the durability of the effects, while long-term maintenance of lifestyle changes is crucial for sustained success. Finally, body composition assessed by BIA, although standardised, remains sensitive to changes in hydration status, which may affect the accuracy of FFM estimates. Given the growing interest in lifestyle interventions in the care of people with type 2 diabetes, further studies with larger sample sizes are warranted.

## Conclusion

These findings suggest that a structured, group-based lifestyle programme delivered by an MDT can be implemented in routine care for people with type 2 diabetes in Poland and may lead to clinically meaningful improvements in glycaemic control and body weight. Greater reductions in weight and BMI among CGM users indicate that intermittent CGM, when integrated into structured education, may provide additional benefit.

## Data Availability

The datasets analysed during the current study are not publicly available due to privacy and ethical restrictions related to participant-level clinical data but are available from the corresponding author on request.

## Abbreviations

95% CI: 95% confidence interval
BIA: Bioelectrical impedance analysis
BMI: Body mass index
C4D: CARE4DIABETES
CGM: Continuous glucose monitoring
COPD: Chronic obstructive pulmonary disease
Cohen’s d: Effect size
DNSG: Diabetes Nutrition Study Group
DSMES: Diabetes self–management education and support
EASD: European Association for the Study of Diabetes
eGFR: Estimated glomerular filtration rate
ESPEN: European Society for Clinical Nutrition and Metabolism
FFM: Fat–free mass
GLP: 1–Glucagon–like peptide–1 (receptor agonists)
GP: General practitioner
HbA1c: Glycated hemoglobin
HDL: C–High–density lipoprotein cholesterol
isCGM: Intermittently scanned continuous glucose monitoring
LDL: C–Low–density lipoprotein cholesterol
M: Mean
MDT: Multidisciplinary team
N: Number of participants / sample size
ns: Not significant
p: p–value
PTD: Polish Diabetes Association (Polskie Towarzystwo Diabetologiczne)
SD: Standard deviation
SGLT: 2–Sodium–glucose cotransporter–2 (inhibitors)
Sig.: Significance
SMBG: Self–monitoring of blood glucose
SMS: Short Message Service
T2DM: Type 2 diabetes mellitus
TIR: Time in range
Δ: Change (delta)
